# Social media analysis of Twitter tweets related to ASD in 2019–2020, with particular attention to COVID-19: topic modelling and sentiment analysis

**DOI:** 10.1186/s40537-022-00666-4

**Published:** 2022-11-25

**Authors:** Luca Corti, Michele Zanetti, Giovanni Tricella, Maurizio Bonati

**Affiliations:** 1 Laboratory for Mother and Child Health, Department of Public Health Istituto Di Ricerche Farmacologiche Mario Negri IRCCS, Via Mario Negri 2, 20156 Milan, Italy; 2 Laboratory Clinical Data Science, Department of Public Health Istituto Di Ricerche Farmacologiche Mario Negri IRCCS, Via Mario Negri 2, 20156 Milan, Italy

**Keywords:** Autism spectrum disorders, Social media, Twitter Messagging, Public health, Sentiment analysis, Content analysis, COVID-19

## Abstract

**Background:**

Social media contains an overabundance of health information relating to people living with different type of diseases. Autism spectrum disorder (ASD) is a complex neurodevelopmental condition with lifelong impacts and reported trends have revealed a considerable increase in prevalence and incidence. Research had shown that the ASD community provides significant support to its members through Twitter, providing information about their values and perceptions through their use of words and emotional stance. Our purpose was to analyze all the messages posted on Twitter platform regarding ASD and analyze the topics covered within the tweets, to understand the attitude of the various people interested in the topic. In particular, we focused on the discussion of ASD and COVID-19.

**Methods:**

The data collection process was based on the search for tweets through hashtags and keywords. After bots screening, the NMF (Non-Negative Matrix Factorization) method was used for topic modeling because it produces more coherent topics compared to other solutions. Sentiment scores were calculated using AFiNN for each tweet to represent its negative to positive emotion.

**Results:**

From the 2.458.929 tweets produced in 2020, 691.582 users were extracted (188 bots which generated 59.104 tweets), while from the 2.393.236 total tweets from 2019, the number of identified users was 684.032 (230 bots which generated 50.057 tweets). The total number of COVID-ASD tweets is only a small part of the total dataset. Often, the negative sentiment identified in the sentiment analysis referred to anger towards COVID-19 and its management, while the positive sentiment reflected the necessity to provide constant support to people with ASD.

**Conclusions:**

Social media contributes to a great discussion on topics related to autism, especially with regards to focus on family, community, and therapies. The COVID-19 pandemic increased the use of social media, especially during the lockdown period. It is important to help develop and distribute appropriate, evidence-based ASD-related information.

## Background

With the spread of social networks and different digital technologies, the way people communicate has undergone profound changes. Social networks are, in fact, valuable sources of information that help involve a large number of individuals in discussions on current issues, and allow people from different cultures to have the opportunity to compare opinions and interests. This exchange of information also makes it possible to spread and elaborate some very important messages in various parts of the world but, above all, allows us to understand the attitudes of individuals towards the issues addressed, obtaining valuable contributions for the promotion of common objectives.

Social media contains an overabundance of health information relating to people living with different type of diseases [1, 2].

Twitter represents one of the leading platforms for political and social activity, and is an effective tool for reflecting and predicting public opinion on different topics [3].

In fact, social media are increasingly important when dealing with health care issues [4]. During the COVID-19 pandemic Twitter experienced a 23% increase in daily use compared to the previous period [5].

Several studies have analyzed the emotional state of people during various lockdowns through the use of twitter [6, 7].

Autism spectrum disorder (ASD) is a complex neurodevelopmental condition with lifelong impacts. Over the last two decades, reported trends in ASD have revealed a considerable increase in prevalence and incidence [8]. ASD is one of the most serious neurodevelopmental conditions in high income countries, with significant caregiver, family, and financial burdens. Overall, estimates range from 0.19/1000 to 11.6/1000 people [9]. Stakeholders within autism spectrum disorder communities use social media to share specific informations and to share ideas [10]. Research had shown that the ASD community provides significant support to its members through Twitter, providing information about their values and perceptions through their use of words and emotional stance [11].

The aim of this study was to analyze the messages posted on this micro blogging platform regarding ASD.

The primary objective was to analyse the topics covered within the tweets and understand the attitude of the various people interested in the topic. In particular, we focused on the discussion of ASD and COVID-19.

The research hypothesis was that social platforms can generate relevant information, almost in real time, capable of contributing and integrating the data collected by national and international statistics centers.

The platform chosen for data collection was Twitter: with approximately 500 million active users worldwide [12], it allows interaction through the publication of short messages (maximum 140 characters) known as tweets. It is believed that the attitudes of individual users are able to significantly influence the opinions and behavior of other individuals in different thematic areas about human activities, events and news spread daily all over the world.

The rest of this paper discusses materials and methods; data analysis; results; discussion and conclusions.

## Materials and methods

### Data extraction

The analysis was carried out on a large dataset created by the collection of information on Twitter, with the aim of characterizing the discussions on social media about ASD, and therefore understanding how the topic is perceived by different users, in order to analyze their behavior.

The data collection process was based on the search for tweets through hashtags and keywords.

A hashtag, represented by the # symbol, is used to indicate keywords and topics on Twitter, allowing users to quickly and easily follow topics of interest. In fact, by clicking on a word in the form of a hashtag in the text of a tweet, all the messages in which this hashtag appears are shown and, if this is a popular hashtag, it represents a trending topic.

The reference terms were extracted, both, following research carried out on the Twitter platform [13, 14], and following the analysis of studies published on different scientific platforms (e.g. PubMed), referring to research activities on the ASD topic. This allowed us to verify the presence or absence of past studies and to align our work with the current studies in the various journals.

Following this research step, and after careful evaluation, the hashtags and keywords identified, referring to the trending topics that generated the largest number of tweets in English on the ASD topic, were the following: # autism; #ASD; #ActuallyAutistic; #AutismSpeaks; #AutismParent; #Autchat; #AutismAwareness; #WorldAutismAwarenessDay; aspergers; autism; autistic; autism sprectrum; Disorder; spectrumDisorder; autism disorder.

These terms were chosen because they were identified as the most representative for conducting a study on the topic of ASD, also using the terms found in the medical subject headings (MeSH) available from the Medline (PubMed) biomedical literature database.

Following an initial survey, other hashtags were, in fact, taken into consideration, but these were subsequently eliminated as their contribution to the topic was limited.

The data collection was performed using web scraping, a technique that allows the collection of unstructured data from the network and its transformation into metadata, which can then be stored and analyzed in a database [15].

To do this, the following steps were followed:

1. The setting up of specific, necessary search parameters was required. The most important was the search term of interest, used to search for tweets, together with the language in which the tweet was written.
2. To perform a more specific search, it was necessary to identify the starting and stopping dates for data collection.
3. After setting up these parameters it was possible to start searching for, and collecting, the actual tweets.
4. A limited set of information was returned for each tweet collected. This information includee the full text of the tweet, the creation date, the number of replies obtained, and the number of retweets obtained.

All subsequent manipulation of the collected data was performed through the use of the Twitter search application programming interface (API), which made it possible to integrate information regarding individual tweets and to obtain all the entities (e.g. hashtags, media, user mentions etc.) making up the tweets.

Once a dataset was available that provided a limited set of data for each single tweet, it was necessary to use the Twitter API to obtain all the information concerning the message. This was made possible by the Twitter API (Get statuses / lookup [16]), thanks to a process known as “rehydration”. By passing the unique identifier of a tweet as a parameter, it was possible to obtain all the information concerning it. After doing this for every tweet in the dataset a larger and more complete dataset was available. The first objective was to identify all users who generated a tweet: in addition to the unique identifier that represents a user, for each user the number of "followers", the number of "friends", and the number of total tweets the user generated and the number of retweets obtained was obtained.

In particular, the number of total tweets for each user was measured as an aggregation of the tweets in the dataset that are traceable to that user. Similarly, the number of retweets and the number of favorites obtained by individuals were calculated. By doing this, a new dataset was created for the bot identification.

### Bot identification

Bots are applications that perform automated activities on the internet [17]. Their use is increasingly widespread on microblogging platforms and, depending on their degree of "evolution", they are able to imitate human behavior thanks to the use of artificial intelligence. A social bot can therefore be defined as an account on a social media that is controlled in whole or in part by software.

As a result, their detection is fundamental, as the activity of a bot is able to trigger the viral spread of false or unreliable news, allowing this information to reach high visibility and increasing the probability of wide-scale sharing by many real accounts.

A web-based program called Botometer was used to identify bots within the dataset created. Users extracted were then ordered in terms of the number of tweets produced and retweets obtained, in order to analyze the major exponents of these two groups (bot and non bot). The hypothesis was that profiles that produced a high number of tweets and retweets were more likely to be identified as bots than profiles that generated few tweets and obtained few retweets.

This screening was used to identify the profiles that generated a large amount of tweets and that were therefore able spread their information within the platform: users who produced a limited number of tweets and who did not obtain a high diffusion were considered "harmless" with regard to the spread of disinformation.

To identify the most active users we considered those who had generated a number of tweets equal to, or greater, than 50 during the year, which, on average, corresponds to about 4 tweets per month.

Furthermore, based on the distribution of the values returned by Botometer, 0,86 was chosen as the threshold: if the value associated with a user was greater than or equal to this threshold then the user was classified as a bot instead of as a normal user [18].

## Data analysis

### Content analysis

Topic modeling is an unsupervised machine learning technique that allows a document to be processed in order to obtain the topics discussed within it. It is an important part of the traditional Natural Processing Approach and thanks to its potential it is possible to obtain semantic relationships between words in clusters of documents: each topic, in fact, is a collection of the most important and representative words for that topic [19].

There are different possible approaches, but the NMF (Non-Negative Matrix Factorization) method was used in this study because it produces more coherent topics compared to other solutions.

NMF is a statistical method to reduce the dimension of the input corpora. It uses the factor analysis method to provide comparatively less weight to the words with less coherence: each of the words in the document are given a weight based on the semantic relationship between the words, but the one with highest weight is considered as the topic for a set of words [20].

Pre-processing was necessary before applying the NMF, however, since tweets often contain grammatically incorrect sentences and non-standard words, as well as abbreviations and phonetic substitutions. To overcome this problem and obtain a correct result, pre-processing allowed us to obtain a standardized form to be used in the NMF model. First of all, disturbing elements such as HTML Tags, Stopwords, Punctuation, White Spaces, URLs, etc. were removed.

Next, the text obtained was normalized through Tokenization, Lemmatization, Stemming, Sentence segmentation, etc., thus obtaining the clean text to be used in the model.

At this point, the dataset included all the tweets divided into topics.

Based on this partition, the analysis focused on identifying the sentiment within each topic: it was important to understand how the particular topic was perceived by various users.

### Sentiment analysis

Sentiment analysis is part of the greater field of text mining, also known as text analysis, which is the process for detecting the sentiment expressed about the topic of interest in a sentence. Sentiment analysis is one field of natural language processing, useful with large amounts of textual information from which is possible to extract general information.

Modern sentiment analysis tools are based on algorithms that can handle huge volumes of information and, when associated with text analysis, sentiment analysis provides interesting information on the opinion of different users who play a fundamental role in this type of analysis.

In fact, it is not enough to know the topic of interest, it is also essential to know what people think about it. This type of classification focuses on the polarity of a certain text, which determines whether the opinion expressed is positive, negative, or neutral [21].

One of the most indirect ways of acquiring textual data is through social media mining, made possible by the use of social media management software with tracking capabilities. Sentiment analysis can be carried out in different ways, using machine learning, statistics, and / or natural language processing (NLP) to find out how people think and feel on a macro scale.

In this study sentiment analysis was based on rule-based sentiment analysis, a method that uses a lexicon, or word-list, where each word is given a score for sentiment and sentences are assessed for overall positivity, neutrality, or negativity using these weightings.

In particular, we used AFiNN, a lexicon that includes 2477 words that are able to analyze the language used in micro blogging platforms such as Twitter. AFiNN uses a scoring system in which each word is assigned a value from −5 (very negative) to + 5 (very positive). Words with a score below − 0.05 are identified as negative, while those with a score above 0.05 are identified as positive [22].

The study was conducted on the tweets produced during the year 2019 and 2020; the goal was to be able to make a comparison between the two years, to identify if and how the revelation of COVID-19 affected the way in which the topic considered was expressed and perceived. The two datasets included, respectively, 2,393,236 and 2,458,929 tweets (of which 73,354 referring to COVID-19).

## Results

### Bot 2019–2020

Starting from the 2.458.929 tweets produced in 2020, 691.582 users were extracted, while from the 2.393.236 total tweets from 2019, the number of identified users was 684.032.

**Fig. 1:**
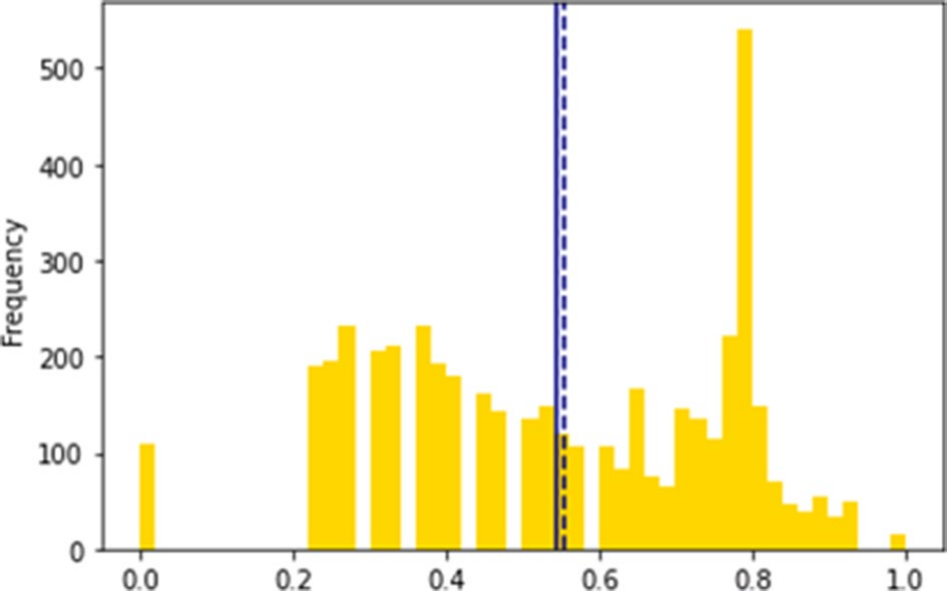
The probability distributions of bots

Referring to the partitions previously explained in the methods, in 2020, from a total of 691.582 single users, 4.913 were identified as “most active” and bot analysis revealed the presence of 188 bots, which generated 59.104 tweets (Fig. 1).

In 2019, on the other hand, from 684.032 total single users, those taken into consideration were 4.675. In this case, the bot analysis identified the presence of 230 bots, although the number of tweets they generated was 50.057.

Following this identification, tweets were divided into two groups: tweets generated by users and tweets generated by profiles that were identified as bots.

**Fig. 2:**
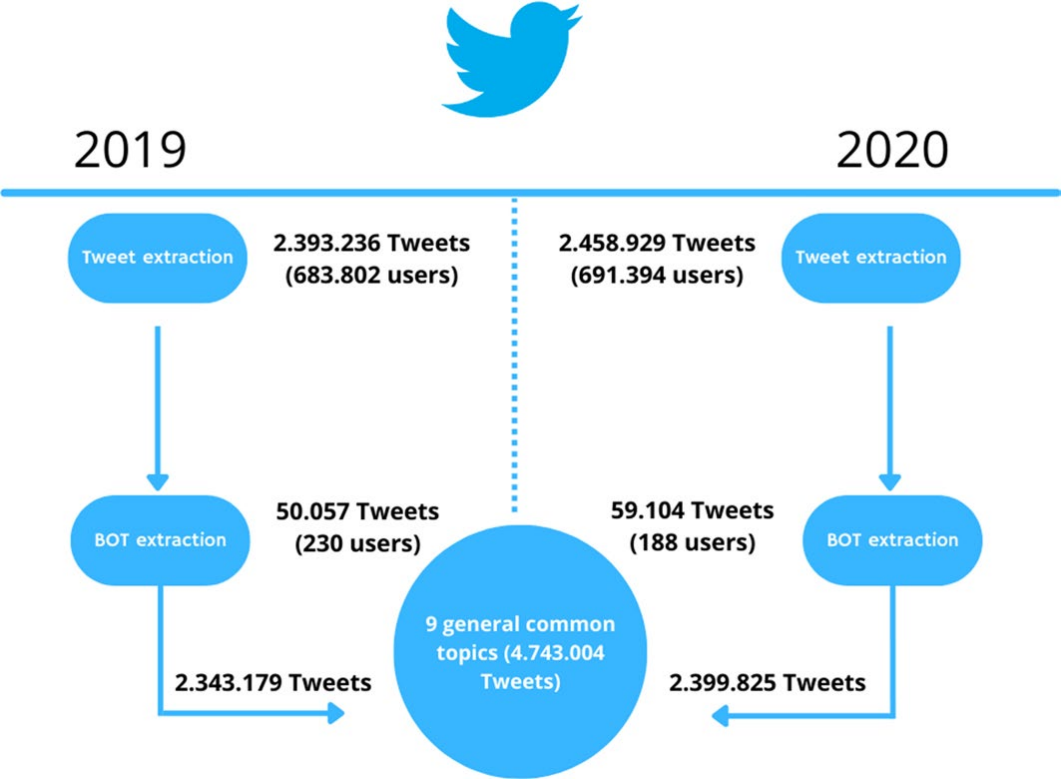
Data flow overview of ASD Tweets in 2019–2020

This partition was carried out in order to perform sentiment analysis on tweets generated by actually existing users (Fig. 2).

### Topic modelling 2019–2020

Topic modeling was carried out on tweets of both 2019 and 2020; this analysis was conducted in order to compare the two reference years to understand if the topics covered were different or similar.

Starting from the 2019 and 2020 tweets, therefore, the top 20 most important topics were considered. This choice was due to the fact that, considering fewer topics, the groups of tweets obtained were too generic, thus introducing important limitations during the final analysis. In fact, when referring to excessively numerous groups noise was introduced and this initially affected the final result, not allowing a clear identification of the most important topics. On the other hand, when considering more than 20 topics the difference between the number of tweets included in the first topic and in the last topic was too wide, making it unnecessary to consider more than 20 topics.

When comparing the topics identified in the two reference years, the main topics covered were found to be very similar to each other. This similarity allowed us to enclose the identified topics in 9 macro groups, based on the subject, as follows:

- Family: families who have children with autism help and support each other. These families need special attention and a different approach than traditional ones. Family activities are carried out with their children and, in some cases, parents express pride concerning their children.
- Community: there is a community where people with autism, both young and older, have the opportunity to express their opinions, fears and feelings. They have the opportunity to compare and support each other because they feel free to talk since autism is a normal topic of conversation. Autism becomes awareness.
- Therapy/Diagnosis: experiences in which autism was diagnosed, as well as some treatments, are discussed in order to relieve stressful and critical situations that may occur. Aspects of Asperger's Syndrome are also discussed.
- Research: research studies carried out on autism spectrum disorder, the symptoms and difficulties are defined.
- Autism Day: World Autism Awareness Day, internationally recognized to encourage awareness of people with autism.
- Negative effects: difficult situations that users with autism have to face (anxiety, mental depression, neurodiversity), which also lead to anger and hatred towards the disease.
- Vaccines: the vaccine issue is cause of conflicting opinions. There are those who claim that vaccines are not a trigger for autism, while others claim the opposite.
- Promotions: websites to raise funds for autistic people, promotion of books and various activities.
- Exceptional events: exceptional events that occurred during the year that are also reported in the news.

This grouping was completed by merging tweets concerning the various macro topics. The individual users who participated in each macro category were also identified, as many users generated tweets belonging to several topics.

Some topics of greatest interest during 2019 were found to have undergone a certain downsizing in favor of other topics that developed a greater interest. Indeed, there was a change in the number of tweets within the various macro categories (Table 1).

**Table 1:** Percentage of tweets in macro groups

Macro groups	2019 (%)	2020 (%)
Family	10	18
Community	46	31
Therapy/Diagnosis	14	22
Research	3	6
Autism Day	5	4
Negative effects	0	4
Vaccines	12	4
Promotions	2	5
Exceptional events	8	6

The same aspect was highlighted with regard to users, who confirm this change in topics, given the number of users who generated tweets within the various classes.

**Fig. 3:**
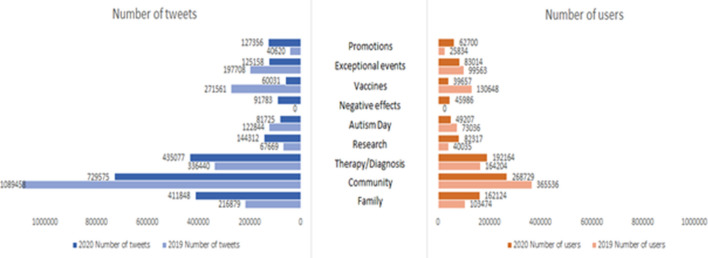
Number of tweets and users in macro groups in 2019 and 2020

Participation depends on the topics covered. There are, in fact, topics that have had a greater impact since they involved a larger number of users and produced a higher flow of tweets than other, less relevant topics (Fig. 3).

This stems from the fact that users actively contributed to the discussions of greatest interest since the desire to express their thoughts and feelings was strong. In particular, many occasions occur in which users agree with a certain topic, but express themselves through negative language that allows them to transmit feelings of anger and frustration due to the various situations they are forced to face.

There are also groups of users who appear to express extremist thoughts about certain situations and who definitively condemn certain identified situations, such as the often inefficient management of the pandemic by their governments.

Although 2020 was a completely exceptional year due to the outbreak of the coronavirus pandemic, the number of tweets that resulted concerning the pandemic was much lower than that of the tweets referring to autism.

This grouping showed the differences between 2019 and 2020. Indeed, significant differences were found regarding some topics, while others resulted more linear.

The "Community" and "Vaccines" topics, for example, showed a drop in interest during 2020, while topics such as "Research" and "Family" had a greater diffusion in 2020.

The "Negative effects" topic was not addressed in 2019: this comes from the fact that during this year no topics related to this macro group were identified, unlike in 2020 where one topic was attributable to this macro group.

It must be emphasized that these changes, which at first glance could be misleading, are not a consequence of the individual groupings into the macro topics carried out, but are due to the fact that the topics covered are much broader. Consequently, to obtain more information, further investigation would be necessary.

The presence of a large amount of tweets, indeed, generated large groups, which would need further analysis, for example through further topic modeling steps, in order to obtain more information about these diversifications.

### COVID-19–ASD tweet

The main focus of this study, however, was to investigate the issue of COVID-19 in relation to ASD. Following the identification of the different topics from 2020, the absence of a single topic about COVID-19 was therefore highlighted. This absence was due to the large amount of tweets extracted and to the fact that the tweets regarding COVID-19 and autism represent a small percentage of the total dataset.

In fact, the results obtained suggested that autism remained the main problem that was being discussed. To jointly investigate ASD and COVID-19, in order to understand how, due to the emergence of the pandemic, the speeches regarding ASD have changed and, above all, to understand what are the main topics within these tweets we identified different keywords relating to COVID-19 and used them to extract tweets concerning autism and COVID-19.

The reference words were: COVID-19; Coronavirus; Pandemic; Quarantine; Lockdown.

**Fig. 4:**
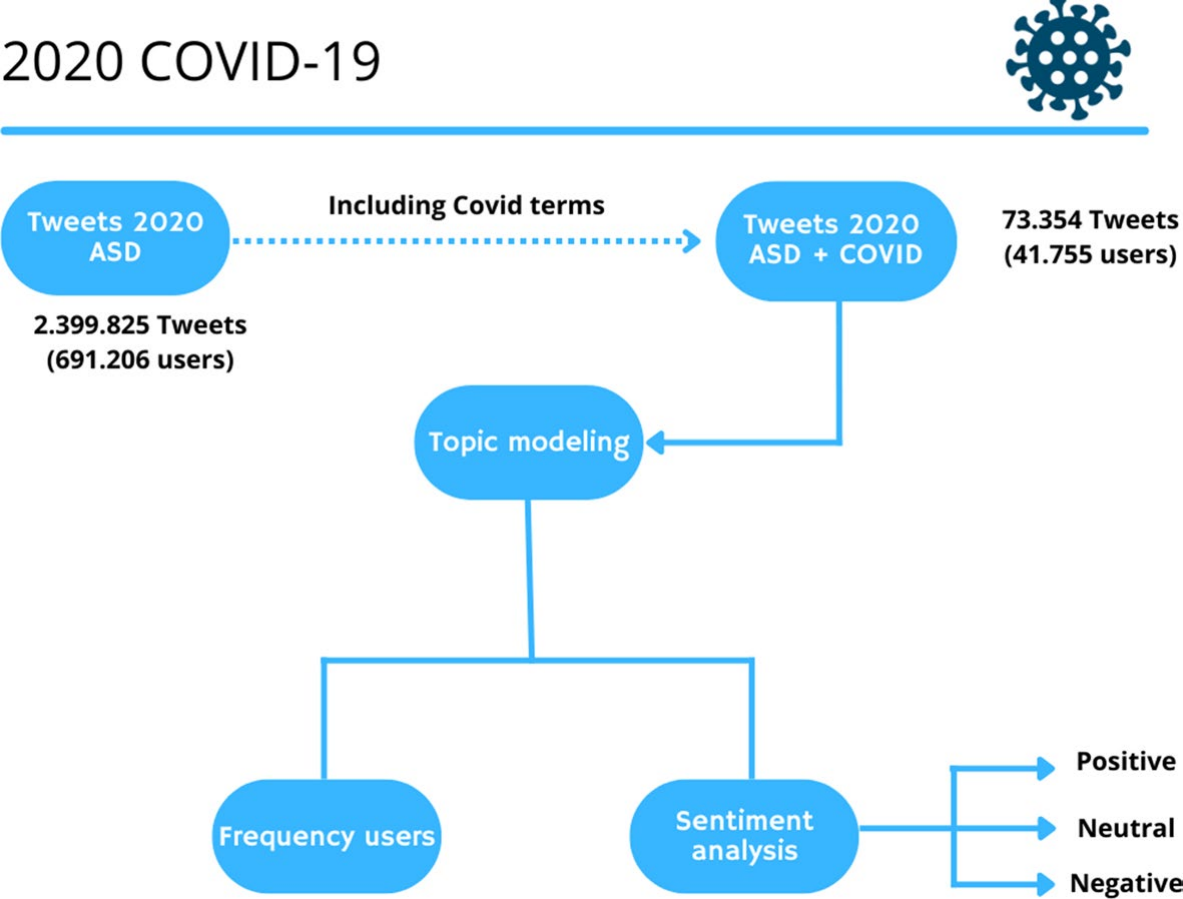
Data flow overview 2020 COVID-19 Tweet

The identified tweets amounted to about 73,354 (approximately 3% of the dataset) (Fig. 4).

This step made it possible to identify tweets concerning COVID-19 that were used in the next phase.

The application of topic modeling on COVID-related tweets revealed its impact on people with ASD, in particular on how they had to face various difficulties throughout the year. One of these concerned the need to change daily habits during the pandemic.

Parents' care toward their children with ASD increased and their management became more complicated: they had to face isolation, which had consequences on autistic people’s mental health, causing a regression in some subjects.

In order to address the burden of isolation, different guidelines and tips were released to help restore people's well-being. In addition, "social pods" were identified as an additional aid in relieving stress and anxiety, allowing people to keep in touch with autistic communities, whose members gravitate towards small groups of friends.

There were also comments regarding vaccines and the delay in their production and distribution, as well as opinions on the expected effects of COVID and criticisms of the political system that managed the pandemic (Table 2).

**Table 2:** Distribution of the number of tweets with relative description, sharing and sentiment analysis

Topic ID	Topic	Description	N (tweet %)	Likes Mean (CI 95%)	Shares Mean (CI 95%)	Negative (%)	Positive (%)	Neutral (%)
1	Lockdown autistic—work—care—going school	Cope with autism during the lockdown: autistic people need care and attention. Parenting is difficult	12,588 (17.16)	17.86 (7.14–28.59)	2.65 (1.41–3.89)	48.7	36	15.3
2	Incredibly confused parenting—support autism families during pandemic	Need for family support to help their autistic children change routines during the pandemic	8,356 (11.40)	6.94 (5.75–8.13)	2.10 (1.77–2.42)	34.1	44.5	21.4
3	BBC news—coronavirus lockdown—autism awareness—lockdown autism—struggle living	BBC news about coronavirus lockdown; mental decline of several autistic people: awareness of autism needed to help those who struggle against COVID-19	7,550 (10.30)	10.42 (6.09–14.74)	1.97 (1.73–2.22)	30.4	48.7	20.9
4	Deal coronavirus—support autistic	Autism and isolation: How coronavirus is affecting kids on the spectrum and their parents. Guide to support the management of patients with autism during the coronavirus pandemic	6,453 (8.80)	11.19 (8.55–13,82)	3.80 (3.05–4.55)	41.6	40.3	18.1
5	coronavirus lockdown—researchers delay—coronavirus outbreak—coronavirus vaccine—coronavirus autism	Impact of the coronavirus outbreak on mental health of autistic people. Vaccine delays	6,140 (8.37)	6.22 (4.40–8.04)	2.39 (2.04–2.74)	35.8	40	24.2
6	autism awareness—crisis—autism & covid	Difficulties experienced by the autism community during the COVID-19 crisis and the impact on their organization: hard working to support and protect community throughout this crisis	5,301 (7.22)	4.25 (3.27–5.24)	1.68 (1.37–1.99)	32.4	39.9	27.7
7	Vaccine—covid vaccine—vaccine autism	Sarcasm on the covid vaccine. Some claim it causes autism	4,921 (6.70)	8.79 (6.89–10.69)	1.71 (1.29–2.12)	60.3	22.2	17.5
8	Italian allowed—play—park—autism allowed	Children with autism allowed to play in Italian park	4,703 (6.41)	7.25 (5.06–9.45)	2.72 (2.09–3.36)	34.9	41.8	23.3
9	Isolation—distancing—masks—autism social	Regression during lockdown of autistic people: "social pods" could alleviate isolation and may work well for the autistic community, whose members gravitate towards small groups of friends	3,374 (4.60)	8.73 (6.23–11.23)	1.99 (1.53–2.46)	31.3	48.6	20.1
10	autism cope—help autistic kids—great tips—tips help—coronavirus quarantine	Tips for helping autistic kids cope with the chaos and uncertainty of coronavirus	2,961 (4.04)	5.11 (3.19–7.04)	2.14 (1.51–2.76)	24.9	54.6	20.5
11	years old boy autistic—lockdown challenges	Boys of different ages with autism facing lockdown challenges in different ways	2,890 (3.94)	14.98 (7.77–22.19)	2.29 (1.57–3.01)	43	39.6	17.4
12	disability autism—mental health—learning disabilities	Guidance on mental health and wellbeing aspects of coronavirus for people with existing mental health difficulties, with learning disability or autism	2,549 (3.47)	6.96 (3.27–10.65)	4.57 (1.53–7.62)	55.2	34	10.8
13	Pharma—genetic—vaccines—end autism—epidemic autism	Someone refers to autism as an epidemic, others say that it is absurd: it is about genetics	2,074 (2.83)	6.56 (4.43–8.68)	1.93 (1.15–2.72)	50.3	25.7	24
14	really badly suffer—autism charity—suffer anxiety depression—depression autism—help retweeting	Autistic people suffering from anxiety and depression by virus and autism. Helping with charity and retweeting	865 (1.18)	4.80 (2.84–6.75)	1.47 (0.93–2.01)	36.6	46.5	16.9
15	learning disabilities—coronavirus rules—disabilities world	Coronavirus rules relaxed for people with autism and learning disabilities	732 (1.00)	6.59 (2.32–10.86)	5.47 (3.49–7.44)	25.5	19.2	55.3
16	People carers—lockdown charities—asking support—disabled customers	Carers struggling to get food during lockdown: different charities asking to support disabled customers	523 (0.71)	3.99 (3.29–4.70)	1.50 (1.19–1.80)	7.3	91.2	1.5
17	BBC news—news coronavirus—autistic support	BBC news about coronavirus and autism. Extra support needed	470 (0.64)	4.85 (3.05–6.65)	3.42 (1.98–4.86)	27.2	61.7	11.1
18	Second wave—public urgently—calling act—impacted autistic—make plan action—coronavirus impacted	Coronavirus has impacted autistic people worse than the rest of the public. Urgent action is needed to have an action plan	364 (0.50)	1.63 (0.83–2.43)	0.85 (0.43–1.28)	85.2	11.5	3.3
19	vaccine manufacturers—asking vaccine—the covid vaxx—weed killer—vaccines glyphosate toxins	New study claims that glyphosate toxins, weed killer and/or aluminium could cause autism. Consumers asking the Vaccine Manufacturers to test the COVID-19 vaccines for glyphosate and other toxins	281 (0.38)	0.54 (0.23–0.86)	0.27 (0.10–0.44)	5.7	90	4.3
20	critical outbreak—political cowardice—covid virus—second wave—diseases	Opinions on the expected effects of covid and people who want to keep isolating themselves until they get the right solution for COVID-19. Criticism to politicians who manage COVID-19	259 (0.35)	1.83 (0.53–3.13)	0.61 (0.22–0.10)	87.6	6.6	5.8
	Total		73,354 (100)					
	p-value			< 0.001	< 0.001			

### Sentiment distribution

Most of the tweets produced in the different topics received on average a greater number of likes compared to the number of retweets.

A like can be seen as a "weaker" form of retweeting, as it allows you to support thoughts, opinions or otherwise express an appreciation of the content of the tweet.

Unlike the like, the retweet generates a sharing of the tweet content and therefore is very important as regards to the dissemination of information.

Although most of the topics report an average of likes far higher than retweets, in other topics these values get closer, thus indicating topics that obtained a greater diffusion by users.

This may depend on the topic, which can be more popular, as in the case of the spreading of news that are shared by users with the aim of transmitting that particular information to other users.

There are also topics in which users have a more extremist behavior, however, and are characterized by a very critical attitude.

The number of positive, negative, and neutral tweets reported for each topic is an aspect that needs attention as it can be misleading. In fact, in this particular situation, the definition of “sentiment” is not necessarily linked to a negative or neutral perception, but it is linked to the fact that some people can be, for example, in agreement with a given argument while being categorized with a negative feeling.

This is due to the fact that within tweets it is possible to find words that express, for example, anger, fear, and worry for a particular situation that the user has to face.

An example is the one concerning the topic in which it is stated that Coronavirus has impacted autistic people worse than the rest of the public and urgent effort to set up an action plan is needed. In this case, most of the tweets were categorized as negative, although users agreed with the statement reported, since they expressed themselves with negative words.

Among all the identified topics, it is possible to identify those that have the highest percentage of tweets with a neutral, negative and positive sentiment.

In general, in the topic with the highest percentage of tweets with neutral sentiment, most of the tweets were mainly about disseminating information in order to reach as many people as possible. Indeed, it is not uncommon to find tweets that report different types of news without adding personal comments or other information.

In the case of the topic with the highest percentage of tweets with negative sentiments, rather, opinions were expressed on the expected effects of COVID-19 and criticisms of the political system that managed the pandemic. In this case, the negative sentiment identified referred to the anger towards COVID-19 and its management and there was therefore a general agreement on the previous statement.

**Fig. 5:**
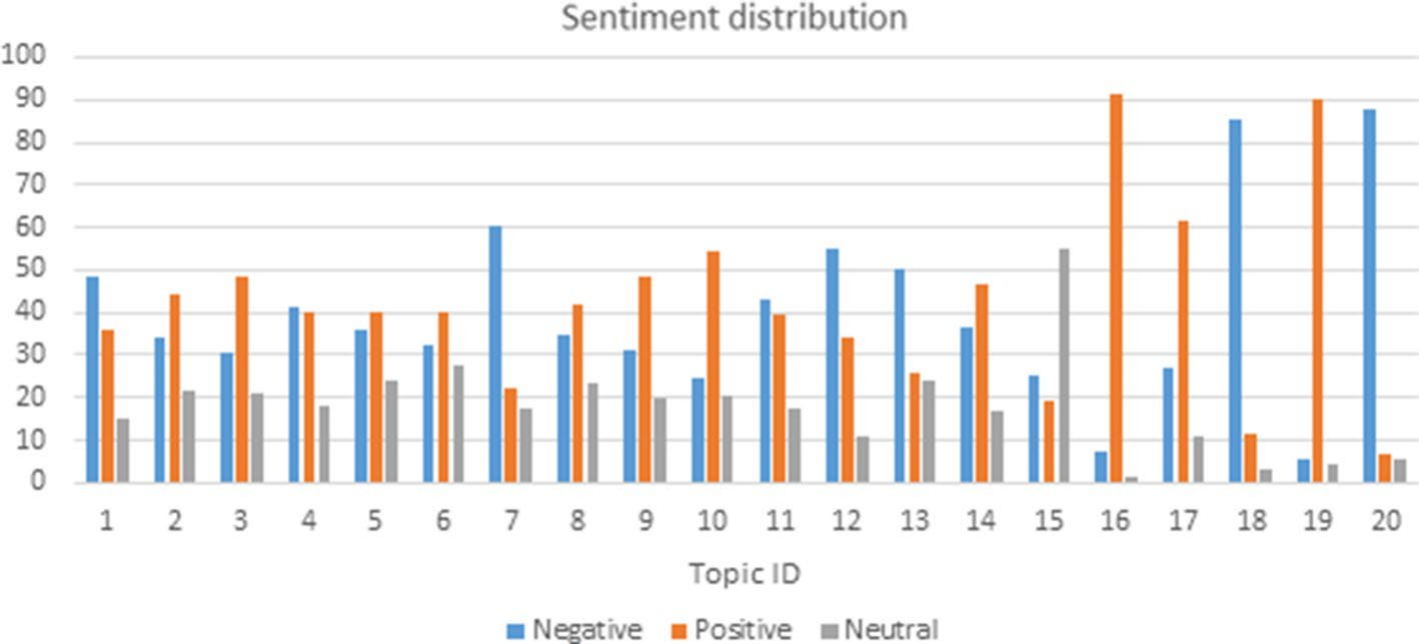
Sentiment distribution in ASD COVID-19 topics during 2020

Finally, even referring to the topic with the highest percentage of tweets with positive sentiments, users are in favor of, and agree, with the statement that it is necessary to provide constant support to people with ASD (Fig. 5).

### User participation

**Fig. 6:**
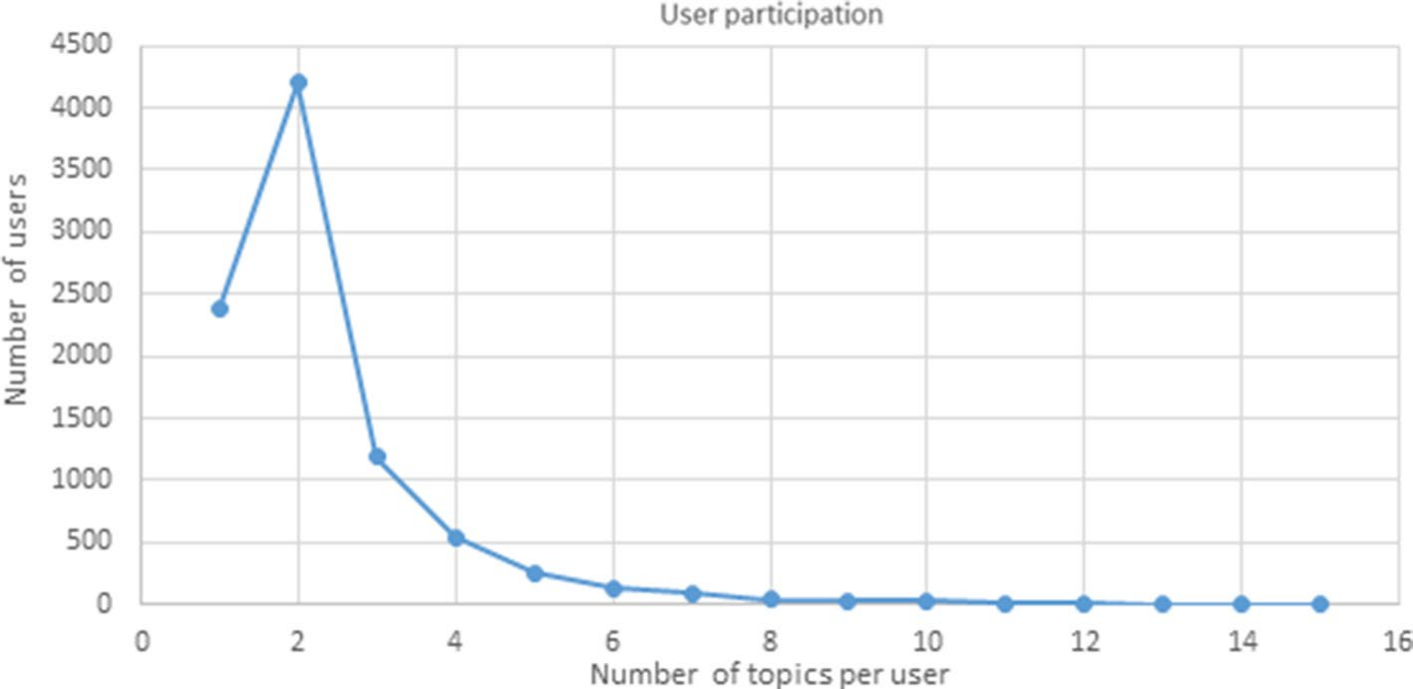
User participation in ASD COVID-19 topics during 2020

After the information flows analysis, the users’ involvement was examined. We expected to find that users were inclined to cover different topics, but, in this case, most users produced tweets concerning only two topics (Fig. 6). More specifically, regardless of the topic, most of the users in the dataset expressed themselves in only one (27%) or two topics (47%). Few users posted multiple tweets on multiple topics (26%), 22% of whom dealt with 3 to 5 topics, and 4% of whom dealt with 6 to 15 topics. It was possible to identify those who dealt with 6 or more topics because they were very few. These profiles were traced back to pages that have a broad interest in the different topics covered and are consequently more inclined to produce content. Indeed, there are organizations that promote the dissemination of news regarding research about autism, and companies whose purpose is to change attitudes and create a society that is also suitable for autistic people. There are also parents with several children with ASD, however, who deal with different topics and provide testimony on their kids' lives, charities for research on autism, and users with autism who share their experiences.

A large number of users who generated few tweets and who obtained few retweets was found, as well users who generated a large number of tweets while obtaining few retweets, thus achieving low diffusion. Unlike these, a few users were able to reach many users with a limited number of tweets,

this is, for example, the case for parents of children with ASD who expressed concerns and/or thoughts that were shared by many people who likely found themselves in the same difficult situation. This led to a few tweets actually going viral.

## Discussion

During the two years analyzed the pandemic accentuated the discussions already dealt with in previous years. The number of tweets and the topics covered are very similar between 2019 and 2020.

We observed that the number of bots is only a small part of the total number of users (2.09% for 2019 and 2.40% for 2020) and generated, respectively, 2.09% and 2.40% of tweets. The total number of COVID-ASD tweets is only a small part of the total dataset (3.05%) and was generated by 6.04% of users. Often, the negative sentiment identified in the sentiment analysis referred to anger towards COVID -19 and its management, while the positive sentiment reflected the necessity to provide constant support to people with ASD. A large number of users in the COVID-ASD tweets generated few tweets, obtained few retweets, and participated in few topics (usually 2 topics).

The main strength is that all tweets produced concerning ASD during the two years were considered in our study and were not further filtered based on the users who produced them (except for bots).

There are some limitations in the tools we used in this study. AFiNN is not able to detect irony and sarcasm, so the sentiment analysis was not flawless [23]. Sentiment analysis also has limitations due to the fact that users and communities are constantly influenced by the social, economic and political environment in which they live and this can influence the content, and therefore the sentiment, of users' tweets [24, 25].

An additional limitation is that only tweets in the English language were considered in this study. This choice was made to reach as many users as possible, since most ome from the United States (69.3 million). When it comes to Twitter, however, the second country with the most users is Japan (50.9 million) [26], so it would be interesting to study opinions and discussions in other languages as well, since tweets in other languages and those from lower-income countries can provide additional insights, especially where the the presence of COVID-19 is high [27].

Another factor that can be considered as a limitation is that most of the tweets produced in the different discussions on Twitter came from a limited number of users who generated the biggest number of tweets [28].

Indeed, only 15% of adults regularly generate content on Twitter, unlike young people and small communities who are more represented than the general population [29], despite the fact that these small communities have been significantly affected by COVID-19 [30, 31].

A large percentage of users therefore remain silent, without producing or retweeting contents, and it would be interesting to study the behavior of these silent majorities.

## Conclusions

In this study, we found that social media, with the example of Twitter, contributes to a great discussion on topics related to autism, especially with regards to focus on family, community, and therapies.

The COVID-19 pandemic further increased the use of social media, especially during the lockdown period. It is therefore important to observe these social networks continuously to try to help limit the dissemination of fake news and help develop and distribute appropriate, evidence-based ASD-related information.

We found that users who have a large influence are only a small percentage of those truly involved and we believe it is important to not limit observations to these few, but to observe all users.

## Data Availability

The datasets generated and/or analysed during the current study are available in the gitlab repository, URL: https://gitlab.com/ITCCC/twitter-conversation-asd-covid-19.

## Abbreviations

ASD: Autism Spectrum Disorders
API: Application programming interface
MeSH: Medical Subject Headings
NMF: Non-Negative Matrix Factorization
